# Endometrial Metaplastic/Reactive Changes Coexistent with Endometrial Hyperplasia and Carcinoma: A Morphological and Immunohistochemical Study

**DOI:** 10.3390/diagnostics12010063

**Published:** 2021-12-28

**Authors:** Antonio Travaglino, Frediano Inzani, Angela Santoro, Damiano Arciuolo, Alessia Piermattei, Sandra Pasquini, Giulia Scaglione, Nicoletta D’Alessandris, Michele Valente, Antonio Raffone, Francesco Fanfani, Gian Franco Zannoni

**Affiliations:** 1 Gynecopathology and Breast Pathology Unit, Department of Woman and Child’s Health and Public Health Sciences, Fondazione Policlinico Universitario Agostino Gemelli IRCCS, 00168 Rome, Italy; antonio.travaglino@unina.it (A.T.); angela.santoro@policlinicogemelli.it (A.S.); damiano.arciuolo@policlinicogemelli.it (D.A.); alessia.piermattei@policlinicogemelli.it (A.P.); sandra.pasquini@policlinicogemelli.it (S.P.); giulia.scaglione@guest.policlinicogemelli.it (G.S.); nicoletta.dalessandris@policlinicogemelli.it (N.D.); michele.valente@guest.policlinicogemelli.it (M.V.); gianfranco.zannoni@unicatt.it (G.F.Z.); 2 Pathology Unit, Department of Advanced Biomedical Sciences, Federico II University of Naples, 80131 Naples, Italy; 3 Department of Life Health and Public Health, Catholic University of the Sacred Hearth, 00168 Rome, Italy; francesco.fanfani@unicatt.it; 4 Division of Gynecology and Human Reproduction Physiopathology, Department of Medical and Surgical Sciences (DIMEC), IRCCS Azienda Ospedaliero-Univeristaria di Bologna. S. Orsola-Malpighi Hospital, University of Bologna, 40138 Bologna, Italy; antonio.raffone2@unibo.it; 5 Gynecologic Oncology Unit, Department of Woman and Child’s Health and Public Health Sciences, Fondazione Policlinico Universitario Agostino Gemelli IRCCS, 00168 Rome, Italy

**Keywords:** metaplasia, reactive, precancer, atypia, morphology

## Abstract

The aim of this study was to assess the relationship between endometrial metaplastic/reactive changes (EMRCs) and endometrial neoplastic lesions. Twenty cases of “simple” (without architecture complexity) EMRCs coexistent with endometrial malignant/premalignant lesions, twenty cases of neoplasia-unassociated EMRCs, and eight cases of complex metaplastic lesions were assessed by immunohistochemistry. EMRCs coexisted with endometrioid carcinoma (*n* = 12), atypical endometrial hyperplasia (*n* = 3), serous carcinoma (*n* = 2), and clear cell carcinoma (*n* = 3). Neoplasia-associated EMRCs showed a mean Ki67 labeling index of 12.6% (range 0–30%); with nuclear atypia in 16/20 (80%) cases; diffuse p16 expression in 15/20 (75%) cases; and heterogeneous ER, PR, and vimentin expression. Compared to the associated neoplasia, EMRCs showed a lower Ki67 expression (*p* < 0.001) and higher p16 expression (*p* < 0.001). No EMRC case showed mitotic activity, PTEN loss, MMR deficiency, nuclear β-catenin, p53-mutant pattern, Napsin A, or AMACR expression. No significant differences were found between neoplasia-associated and neoplasia-unassociated EMRCs. Complex metaplastic lesions showed a lower Ki67 expression than EMRCs (*p* = 0.044) and PTEN loss in 5/8 cases, even in the absence of nuclear atypia. In conclusion, neoplasia-associated simple EMRCs may show evident atypia and a worrisome immunophenotype, but no data support their involvement in endometrial carcinogenesis. Architectural complexity appears as a crucial factor to identify precancerous lesions.

## 1. Introduction

The uterine endometrium is a unique tissue undergoing different phases of hormonal stimulation and may show several different types of altered differentiation, such as tubal, squamous, morular, and mucinous. A peculiar subgroup of endometrial metaplastic change, referred to as “reactive changes”, is characterized by cytoplasmic enlargement with clearing or eosinophilia, hobnail, or pseudopapillary appearance, and is typically found in the presence of ischaemia, desquamation, or hormonal imbalance [1,2]. Reactive changes may also be found admixed with other types of metaplasia [3,4]. These endometrial metaplastic/reactive changes (EMRCs) rarely show cytological atypia, in the form of nuclear enlargement with dispersed chromatin or hyperchromasia, nuclear pleomorphism, and evident nucleoli [3,4,5]. Furthermore, EMRCs are often found in association with endometrial carcinomas [1]. These features have prompted researchers to assess whether some EMRCs may represent premalignant lesions. It has been suggested that the premalignant potential of EMRCs lies in architectural complexity, such as glandular crowding and papillation [6,7,8,9,10,11]; on the other hand, “simple” EMRCs have been regarded as benign reactive changes [3,4,12,13,14,15]. However, to the best of our knowledge, no study assessed whether simple EMRCs associated with endometrial carcinoma may represent premalignant lesions. In the last decades, the emergence of new possible endometrial precancers devoid of architectural complexity makes it mandatory to assess the malignant potential of simple lesions [5,16,17,18]. The aim of this study was to assess the relationship between EMRCs and endometrial neoplastic lesions. This was carried out by (i) reviewing the morphological and immunohistochemical features of simple EMRCs associated with endometrial neoplasia, (ii) comparing EMRCs to the associated neoplasia and the background endometrium, and (iii) comparing neoplasia-associated EMRCs to neoplasia-unassociated EMRCs and to complex metaplastic lesions.

## 2. Materials and Methods

### 2.1. Case Selection

All cases of EMRCs coexistent with endometrial neoplasia (atypical hyperplasia or carcinoma of any type) and diagnosed in the April–September 2021 period were included. EMRCs were defined as endometrial glandular and/or surface epithelium showing cytoplasmic enlargement with clearing or eosinophilia, hobnail cells, cellular tufting, or pseudopapillation (i.e., papillae lacking fibrovascular core); nuclear atypia was assessed based on nuclear enlargement and/or pleomorphism, loss of polarity, and evident nucleoli. Cases showing architectural complexity, such as glandular crowding and true papillation, were excluded. Eight cases of complex metaplastic lesions and 20 cases of EMRCs with no associated neoplasia, diagnosed in the same period, were included as the controls. Only hysterectomy specimens were included. All cases were reviewed by a panel of five pathologists with expertise in gynecological pathology (A.T., A.S., D.A., F.I., and G.F.Z.).

### 2.2. Immunohistochemistry

Immunohistochemistry was performed according to previously described methods [19] and involved prediluited antibodies against PTEN (clone 6H2.1), vimentin (clone V9), P504S/Alpha-methylacyl-CoA racemase (AMACR) (clone 13H4) (Agilent Dako, Santa Clara, CA, United States), MLH1 (clone ESO5), MSH2 (clone 79H11), MSH6 (clone EP49), PMS2 (clone EPS1), p53 (clone Do-7), p16 (clone 6H12) (Leica Biosystems, Wetzlar, Germany), β-catenin (clone 14), estrogen receptor (ER) (clone SP1), progesterone receptor (PR) (clone 1E2), Ki67 (clone 30-9), Napsin A (clone MRQ-60) (Roche, Basel, Switzerland). ER and PR expression were categorized as follows: 3 (strongly and diffusely positive), 2 (strong and focal or diffuse and weak), 1 (weak and focal), or 0 (absent). Ki67 was quantified based on a labeling index (LI, i.e., the percentage of positive nuclei). The PTEN and MMR expressions were dichotomized as “retained” vs. “lost” [19,20]. The β-catenin expression was dichotomized as “membrane” vs. “nuclear” [21]. p53 was dichotomized as “mutant-pattern” (overexpression in >80% of cells, complete absence, or cytoplasmic expression) vs. “wild-type (wt)-pattern” (focal expression or diffuse expression with variable intensity) [22]; the wt pattern was further subdivided into “wt-low” (expression in <5% of cells), “wt-intermediate” (expression in 5–50% of cells), and “wt-high” (expression in >50% of cells). P16 expression was categorized as “diffuse”, “patchy”, or “negative”; a uniformly strong and diffuse expression of p16 was labeled “block-type” [23]. Vimentin expression was categorized as “positive”, “heterogeneous”, or “negative”. Napsin A and AMACR expression were categorized as “positive” or “negative”. Evaluation of the immunohistochemistry was performed by five pathologists (A.T., A.S., D.A., F.I., and G.F.Z.) at a multi-headed microscope.

### 2.3. Data Analysis

Dichotomous variables were compared by using Fisher’s exact test, while continuous variables were compared by using Student’s T test, with a significant *p*-value < 0.05. Data analysis was performed using Statistical Package for Social Science (SPSS) 18.0 package (SPSS Inc., Chicago, IL, USA).

## 3. Results

**Figure 1:**
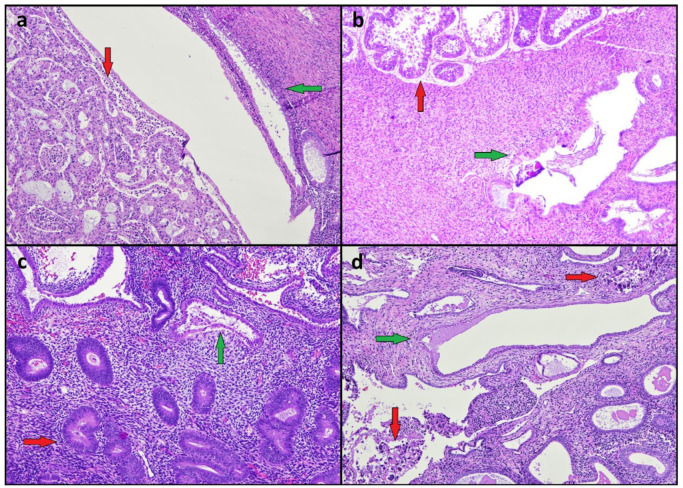
Endometrial metaplastic/reactive changes (green arrows) associated with endometrial neoplasia (red arrows). (**a**) Endometrioid carcinoma. (**b**) Serous carcinoma. (**c**) Atypical endometrial hyperplasia. (**d**) Clear cell carcinoma.

**Figure 2:**
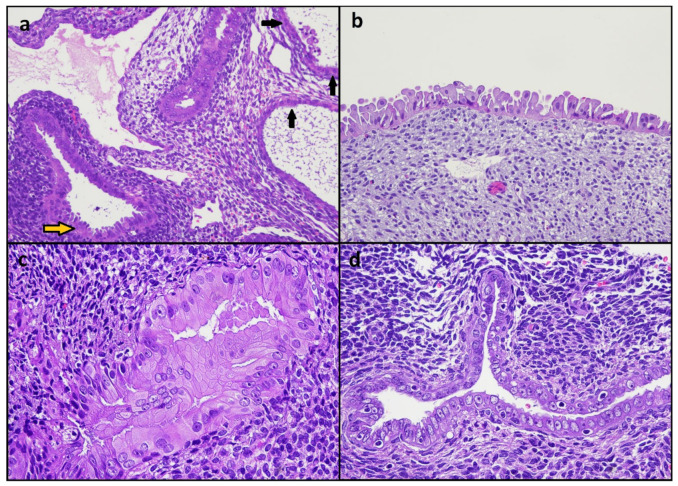
Morphologic features of endometrial metaplastic/reactive changes. (**a**) Eosinophilic changes combined with hobnail (yellow arrow) and ciliated (black arrows) changes. (**b**) Hobnail changes. (**c**,**d**) Cytological atypia with nuclear enlargement, dispersed chromatin, and evident nucleoli.

Twenty EMRCs associated with endometrioid carcinoma (*n* = 10), atypical endometrial hyperplasia (*n* = 5), clear cell carcinoma (*n* = 3), and serous carcinoma (*n* = 2) were identified (Figure 1). EMRCs showed a combination of hobnail, eosinophilic, and ciliated features. The presence of nuclear atypia was determined by consensus in 16/20 (80%) cases (Figure 2); the assignment of atypia according to each observer is shown in Table 1. Mitotic activity was not observed in any EMRC. The expression of ER and PR in EMRCs was heterogeneous; the percentage of cases with a weak/negative ER expression (score 1–2) did not significantly differ between EMRCs (8/20, 40%) and associated neoplasia (6/20, 30%) (*p* = 0.741); PR showed a score 1–2 in 14/20 EMRCs (70%) and 9/20 associated neoplasia (45%), with no significant difference (*p* = 0.200).

**Figure 3:**
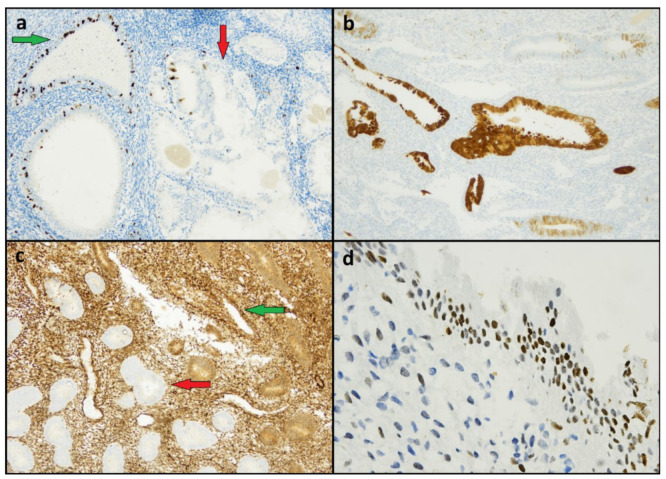
Immunohistochemical features of endometrial metaplastic/reactive changes. (**a**) A case of metaplastic/reactive changes (green arrow) with a Ki67 LI higher than in the coexistent endometrioid carcinoma (red arrow). (**b**) Diffuse and strong p16 expression. (**c**) PTEN expression retained in metaplastic/reactive changes (green arrow) and lost in atypical endometrial hyperplasia (red arrow). (**d**) Wild-type p53 expression with positivity in >50% of the cell nuclei, mimicking a mutant-type pattern.

**Figure 4:**
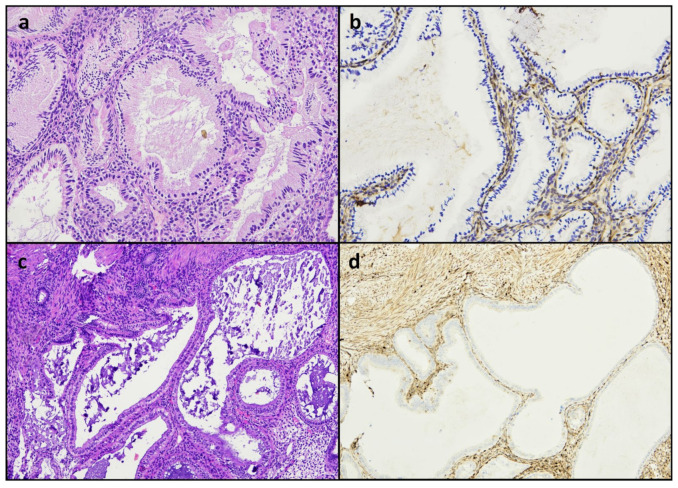
Loss of PTEN expression in complex mucinous metaplasia (**a**,**b**) and complex tubal metaplasia (**c**,**d**) in the absence of evident nuclear atypia.

Ki67 levels were also heterogeneous, with LI varying from 0% to 30%; the mean LI level was significantly lower in EMRC than in associated neoplasia (mean Ki67 LI: 12.6% vs. 39.2%; *p* < 0.001); in two cases, the Ki67 LI of EMRCs was equal to or higher than that of the coexistent neoplasia (Figure 3a). Vimentin expression in EMRCs was heterogeneous in most cases (11/20, 55%), with no significant differences between EMRCs and associated neoplasia (*p* = 1). Fifteen out of twenty EMRCs (75%) showed a diffuse expression of p16, out of which five (25%) were block-type (Figure 3b); a diffuse p16 expression was significantly more common in EMRCs than in the associated neoplasia (3/20, out of which 2 were block-type) (*p* < 0.001). No case of EMRC shared the immunohistochemical markers of the associated neoplasia, including PTEN loss (*n* = 10) (Figure 3c), MMR deficiency (*n* = 4), p53 mutant-type expression (*n* = 4), β-catenin nuclear expression (*n* = 2), Napsin A positivity (*n* = 3), and AMACR positivity (*n* = 2). However, two EMRC cases showed a p53 expression in >50% of cells, mimicking a wild-type pattern (Figure 3d). Moreover, EMRCs showed a higher p53 expression than the associated neoplasia in six cases, a similar expression in nine cases, and a lower expression in only one case. No differences were found between neoplasia-associated EMRCs and neoplasia-unassociated EMRCs. Among eight complex metaplasia cases, five showed a loss of PTEN expression, even in the absence of evident nuclear atypia (Figure 4); complex metaplasias showed a lower Ki67 (7.2%) LI than simple EMRCs (*p* = 0.044); no complex metaplasia case showed p16 block-type expression. The immunohistochemical features of all the included specimens are reported in Table 2 and Table 3.

## 4. Discussion

This study showed that neoplasia-associated EMRCs often display nuclear atypia with a diffuse p16 expression, a p53 expression that may mimic a mutant pattern, and a variably increased Ki67 LI; however, these EMRCs showed neither a mitotic activity nor any marker of endometrial carcinogenesis. Moreover, neoplasia-associated EMRCs did not differ from neoplasia-unassociated EMRCs. On the other hand, complex metaplastic lesions showed PTEN loss, even in the absence of evident nuclear atypia and of a high Ki67 expression.

The wide spectrum of morphologic alterations encountered in endometrial pathology has long since caused diagnostic issues. The first study that addressed the issue of endometrial metaplasia was conducted by Hendrickson and Kempson in 1980. The authors reported several different metaplastic changes that might mimic malignancy; they proposed classifying endometrial metaplasia into seven categories: morular/squamous, papillary, ciliated/tubal, eosinophilic, mucinous, hobnail, and clear cell [24]. Subsequent studies have shown than not all these changes have the same significance. In fact, morular/squamous, tubal, and mucinous metaplasia may be regarded as “true” metaplasia, in the sense that they seem to reflect a transdifferentiation towards another type of epithelium. On the other hand, papillary (subsequently called “syncytial-papilllary”), eosinophilic, hobnail, and clear cell metaplasia have been regarded as “reactive changes”, i.e., non-specific changes reflecting tissue damage and reparation [1]. Reactive changes may often superimpose to true metaplastic changes [3,4]. For instance, in our series, we found a combination of ciliated, hobnail, and eosinophilic changes in most cases, and therefore we adopted the term “EMRC” to define these lesions.

Previous studies assessed the association between EMRCs and carcinogenesis. EMRCs may superimpose to atypical hyperplasia and endometrioid carcinoma; in these cases, architectural complexity is crucial to identify premalignant/malignant lesions [25]. Regarding simple EMRCs, several studies have supported that they are reactive/degenerative changes devoid of a premalignant potential [3,4,12,13,14,15]. However, to our knowledge, no study specifically assessed EMRCs that coexist with endometrial carcinoma. Such a topic may be of value, as not all endometrial carcinoma precursors are associated with architecture complexity [5,16,17]. This is especially true for non-endometrioid carcinoma and, possibly, for a subset of endometrioid carcinomas that arise in atrophic endometrium [5,16,17,18].

We found that neoplasia-associated EMRCs can show evident nuclear atypia with nucleomegaly, pleomorphism, and nucleolation; such atypia might rise the suspicion of serous carcinoma. This issue has prompted several authors to assess immunohistochemical markers to differentiate between the two entities. While most authors have reported that the immunophenotypes of EMRCs and serous carcinoma are completely different, other authors have reported that it is overlapping [3,4,12,13,14,15]. In our series, we found that the mean Ki67 LI was significantly lower in EMRCs than in coexistent neoplasia. However, the Ki67 values in EMRCs were highly variable, with a mean value >10%; in two cases, the Ki67 LI of EMRCs was equal or even higher than the coexistent neoplasia. The expression of p16 was diffuse in 75% of cases (often with a block-type pattern similar to that observed in serous carcinoma), while most associated neoplasias showed a patchy pattern. The expression of p53 was wild-type in all cases, while serous carcinomas and 2/3 clear cell carcinomas showed a p53-abnormal pattern; however, a minority of cases showed p53 positivity in >50% of the cell nuclei, possibly raising the concern of a p53-mutant pattern.

Therefore, EMRCs may show a combination of nuclear atypia and altered immunophenotyped, which may be worrisome to pathologists; this highlights that immunohistochemistry should be interpreted carefully in these lesions. However, mitotic activity was not observed in any EMRC. Furthermore, we assessed several markers of endometrial carcinoma, including PTEN loss MMR deficiency, nuclear β-catenin expression (which are common in atypical hyperplasia and endometrioid carcinoma), p53-mutant-pattern (a marker of serous carcinoma), Napsin A, and AMACR positivity (confirmatory markers of clear cell carcinoma) [2], and none of them were found in EMRCs. In addition, no morphological or immunophenotypical difference was detected between neoplasia-associated and neoplasia-unassociated EMRCs. In contrast, complex metaplastic lesions showed PTEN loss in most cases, even in the absence of marked nuclear atypia and despite showing a Ki67 LI lower than in EMRCs. These findings strengthen the idea that architectural complexity is a crucial factor to define the premalignant potential of EMRCs. Remarkably, the diagnostic criteria of atypical endometrial hyperplasia no longer require an evident atypia, but it is sufficient that the lesion is cytologically different from the background endometrium; instead, the presence of architectural complexity in the form of glandular crowding is a necessary feature [2]. As EMRCs are cytologically altered by definition, it might be appropriate to consider complex EMRCs as analogous to atypical hyperplasia.

It should be remarked that EMRCs may be heterogeneous with regard to morphological and immunophenotypical features. For instance, immunohistochemical markers such as p16, p53, and Ki67 may be highly expressed in some glands and not in other ones within the same EMRC area. Such heterogeneity may be crucial to differentiate EMRCs from malignant/premalignant lesions. Therefore, submitting additional sections can be helpful when only little foci of EMRCs are observed and the morphological/immunophenotypical features are worrisome. The submission of the entire endometrial cavity appears appropriate in cases of complex metaplastic lesions, given their analogies with atypical endometrial hyperplasia.

The main limitation of our study is the lack of follow-up data, as all included specimens were derived from hysterectomies. Determining the biological behavior of EMRCs clearly is impossible for cases that already have a coexistent neoplasia. Regarding neoplasia-unassociated EMRCs, long-term follow-up data from the literature suggest that they do not evolve into overt premalignant/malignant lesions [3]. Further studies are necessary to identify endometrial premalignant lesions devoid of architectural complexity and to define how they should be diagnosed.

## 5. Conclusions

EMRCs that accompany endometrial carcinoma and AEH may show evident nuclear atypia and an aberrant phenotype that might mimic serous carcinoma; however, the lack of mitotic activity and of carcinogenesis-related markers and the similarity with neoplasia-unassociated EMRCs support that they are benign reactive change. By contrast, complex metaplastic lesions often show PTEN loss, even in the absence of evident atypia and of increased proliferation, supporting that architectural complexity is a crucial factor to define the premalignant potential of EMRCs. Further studies are encouraged in this field, with particular regard to the precursors of non-endometrioid carcinomas.

## Figures and Tables

**Table 1:** Assignment of nuclear atypia according to each observer.

Case No.	Presence of Atypia					
	AT	FI	AS	DA	GFZ	Consensus
1	yes	yes	yes	yes	yes	yes
2	yes	no	no	yes	yes	yes
3	yes	yes	yes	yes	yes	yes
4	yes	yes	yes	yes	yes	yes
5	no	no	no	yes	no	no
6	yes	yes	yes	yes	yes	yes
7	yes	yes	yes	yes	yes	yes
8	no	no	no	no	no	no
9	yes	no	yes	no	yes	yes
10	yes	yes	yes	yes	yes	yes
11	yes	yes	yes	yes	yes	yes
12	yes	yes	yes	yes	yes	yes
13	no	yes	yes	yes	yes	yes
14	no	no	no	no	no	no
15	yes	yes	yes	yes	no	yes
16	no	no	no	no	no	no
17	yes	yes	yes	yes	yes	yes
18	yes	yes	yes	yes	yes	yes
19	yes	no	yes	no	yes	yes
20	yes	yes	yes	yes	yes	yes

**Table 2:** Summary of immunohistochemical results (part 1).

Group	Sample Size	Ki67 LI Mean (Range)	ER				PR				p16				Vimentin		
			1	2	3	4	1	2	3	4	Neg	Patchy	Diffuse	Block	-	+/-	-
**Neoplasia-associated EMRCS**	**20**	**12.6% (0–30%)**	**0**	**8**	**8**	**4**	**6**	**8**	**3**	**3**	**0**	**5**	**10**	**5**	**3**	**11**	**6**
-with EC	12	12.9% (0–30%)	0	4	5	3	5	2	3	2	0	2	8	2	1	7	5
-with AEH	3	5.7% (2–10%)	0	2	1	0	0	3	0	0	0	1	1	1	0	3	0
-with SC	2	12.5% (5–20%)	0	1	0	1	1	0	0	1	0	0	0	2	1	1	0
-with CCC	3	18.3% (10–30%)	0	1	2	0	0	3	0	0	0	2	1	0	1	1	1
**Endometrial neoplasia**	**20**	**39.2% (8–90%)**	**4**	**2**	**4**	**10**	**4**	**5**	**5**	**6**	**0**	**17**	**1**	**2**	**4**	**15**	**9**
-EC	12	30.7% (8–70%)	0	1	4	7	0	4	5	3	0	12	0	0	2	12	6
-AEH	3	35% (20–50%)	0	0	0	3	0	0	0	3	0	3	0	0	0	0	3
-SC	2	85% (80–90%)	1	1	0	0	1	1	0	0	0	0	0	2	0	2	0
-CCC	3	46.7% (40–60%)	3	0	0	0	3	0	0	0	0	2	1	0	2	1	0
**Neoplasia-unassociated EMRCs**	**20**	**12.2% (0–50%)**	**0**	**4**	**10**	**6**	**4**	**7**	**6**	**3**	**1**	**8**	**8**	**3**	**4**	**8**	**8**
**Complex metaplasias**	**8**	**7.2% (0–12%)**	**0**	**1**	**3**	**4**	**0**	**3**	**4**	**1**	**0**	**3**	**5**	**0**	**0**	**6**	**2**

**EC**: endometrioid carcinoma; **AEH**: atypical endometrial hyperplasia; **SC**: serous carcinoma; **CCC**: clear cell carcinoma.

**Table 3:** Summary of immunohistochemical results (part 2).

Group	MMR Loss	PTEN Loss	β-Catenin Nuclear	Napsin+/ AMACR+	p53			
					Wt-Low	Wt-Intermediate	Wt-High	Mutant Pattern
**Neoplasia-associated EMRCS**	**0**	**0**	**0**	**0**	**9**	**9**	**2**	**0**
-with EC	0	0	0	0	7	4	1	0
-with AEH	0	0	0	0	0	2	1	0
-with SC	0	0	0	0	0	2	0	0
-with CCC	0	0	0	0	1	2	0	0
**Endometrial neoplasia**	**4**	**10**	**2**	**3**	**7**	**8**	**1**	**4**
-EC	4	7	2	0	5	7	0	0
-AEH	0	1	0	0	1	1	0	0
-SC	0	0	0	0	0	0	0	2
-CCC	0	2	0	3	0	0	1	2
**Neoplasia-unassociated EMRCs**	**0**	**0**	**0**	**0**	**10**	**6**	**4**	**0**
**Complex metaplasias**	**0**	**5**	**0**	**0**	**4**	**4**	**0**	**0**

**EC**: endometrioid carcinoma; **AEH**: atypical endometrial hyperplasia; **SC**: serous carcinoma; **CCC**: clear cell carcinoma.

## Data Availability

Additional data are available from the corresponding author upon reasonable request.
